# Clinical implications of cefoperazone–sulbactam MIC values in *Pseudomonas aeruginosa* bloodstream infections: a multicentre study

**DOI:** 10.1093/jacamr/dlag073

**Published:** 2026-05-11

**Authors:** Shian-Sen Shie, Ting-Shu Wu, Feng-Yee Chang, Ya-Sung Yang, Tsung-Ta Chiang, Mao-Wang Ho, Chia-Hui Chou, Po-Liang LU, Shang-Yi Lin, Fu-Der Wang, Hung-Jen Tang, Zhi-Yuan Shi, Yin-Ching Chuang, Jien-Wei Liu

**Affiliations:** Division of Infectious Diseases, Department of Internal Medicine, Chang Gung Memorial Hospital, Taoyuan, Taiwan; Division of Infectious Diseases, Department of Internal Medicine, Chang Gung Memorial Hospital, Taoyuan, Taiwan; Division of Infectious Diseases and Tropical Medicine, Department of Internal Medicine, Tri- Service General Hospital, National Defense Medical Center, Taipei, Taiwan; Division of Infectious Diseases and Tropical Medicine, Department of Internal Medicine, Tri- Service General Hospital, National Defense Medical Center, Taipei, Taiwan; Institute of Preventive Medicine, National Defense Medical Center, Taipei, Taiwan; Division of Infectious Diseases and Tropical Medicine, Department of Internal Medicine, Tri- Service General Hospital, National Defense Medical Center, Taipei, Taiwan; Division of Infectious Diseases, Department of Internal Medicine, China Medical University Hospital, Taichung, Taiwan; Division of Infectious Diseases, Department of Internal Medicine, China Medical University Hospital, Taichung, Taiwan; Division of Infectious Diseases, Department of Internal Medicine, Kaohsiung Medical University Hospital, Kaohsiung, Taiwan; Center for Liquid Biopsy and Cohort Research, Kaohsiung Medical University, Kaohsiung, Taiwan; College of Medicine, Kaohsiung Medical University, Kaohsiung, Taiwan; Division of Infectious Diseases, Department of Internal Medicine, Kaohsiung Medical University Hospital, Kaohsiung, Taiwan; College of Medicine, Kaohsiung Medical University, Kaohsiung, Taiwan; Department of Laboratory Medicine, Kaohsiung Medical University Hospital, Kaohsiung Medical University, Kaohsiung, Taiwan; Division of Infectious Diseases, Department of Internal Medicine, Taipei Medical University Hospital, Taipei, Taiwan; Institute of Medical and Public Health, National Yang-Ming Chiao-Tung University, Taipei, Taiwan; Department of Medicine, Chi Mei Medical Center, Tainan, Taiwan; Department of Internal Medicine, Taichung Veterans General Hospital, Taichung, Taiwan; Department of Medicine, Chi Mei Medical Center, Tainan, Taiwan; Division of Infectious Diseases, Department of Internal Medicine, Kaohsiung Chang Gung Memorial Hospital, Kaohsiung, Taiwan; Chang Gung University College, School of Medicine, Taoyuan, Taiwan

## Abstract

**Background:**

Cefoperazone/sulbactam (CPZ/SUL, 1:1) is used for *Pseudomonas aeruginosa* bloodstream infections (BSIs); however, its clinical minimum inhibitory concentration (MIC) breakpoint remains undefined. This study aimed to evaluate the association between CPZ/SUL MIC values and clinical outcomes.

**Methods:**

We retrospectively analysed 122 adults with *P. aeruginosa* BSIs treated with CPZ/SUL (1:1) for ≥3 days across eight Taiwanese medical centres (July 2017−May 2024). MICs were determined using agar dilution, and outcomes were categorized as favourable (cure/improvement) or poor (failure/mortality). Isolate susceptibilities to other agents were also evaluated.

**Results:**

Overall, 76.2% achieved favourable outcomes. Patients with MICs ≤16 mg/L had significantly higher favourable rates than those with MICs ≥32 mg/L (81.0% versus 54.5%; *P* = 0.013). This association remained significant across all hierarchical models, including the fully adjusted model (aOR 2.74, 95% CI 1.44–5.25). Pneumonia-associated BSIs had lower favourable outcomes (64.1%). Among all isolates, 82% exhibited MICs ≤16 mg/L. Carbapenem-resistant *P. aeruginosa* (CRPA) accounted for 18% of cases, with 59.1% showing favourable responses to CPZ/SUL.

**Conclusions:**

Lower CPZ/SUL MIC values were independently associated with more favourable clinical outcomes in patients with *P. aeruginosa* BSIs. These findings support the clinical relevance of MIC stratification and may inform antimicrobial stewardship and individualized treatment decisions.

## Introduction

Cefoperazone-sulbactam (CPZ/SUL) represents a β-lactam/β-lactamase inhibitor combination considered a therapeutic option for *Pseudomonas aeruginosa* bloodstream infections (BSIs), particularly in certain Asian regions such as Taiwan where its 1:1 formulation is clinically available. *P. aeruginosa* is a pathogen notorious for healthcare-associated infections in immunocompromised patients, often leading to substantial morbidity and mortality.^1^ BSIs caused by *P. aeruginosa* carry particularly high mortality rates.^2,3^ The emergence of multidrug-resistant (MDR) *P. aeruginosa* in recent years has further exacerbated the challenge, drastically limiting available treatment options and emphasizing the urgent need for effective therapeutic strategies.^4^

Although the Clinical and Laboratory Standards Institute (CLSI) provides minimum inhibitory concentration (MIC) breakpoints for cefoperazone, these are established for Enterobacteriaceae and other non-Enterobacterales, but do not encompass *P. aeruginosa.*^5^ Moreover, specific interpretive criteria for the CPZ/SUL combination, particularly the 1:1 formulation, remain undefined. Currently, available data on the antimicrobial spectrum and MIC values of CPZ/SUL against clinically relevant bacterial isolates are predominantly derived from studies using the 2:1 formulation, which may limit their clinical applicability.^6–8^ Although sulbactam provides limited inhibition against the β-lactamases typically produced by *P. aeruginosa*, the increased proportion of sulbactam in the 1:1 formulation may still confer partial synergistic effects through modest β-lactamase inhibition and pharmacodynamic interaction, thereby enhancing the overall activity of cefoperazone. Previous *in vitro* studies have also demonstrated such additive or synergistic effects between cefoperazone and sulbactam against *P. aeruginosa* strains.^9,10^ In Taiwan, CPZ/SUL (1:1) is frequently used for treating *P. aeruginosa* infections due to its broader availability and demonstrated clinical efficacy.^11^ However, a paucity of published data evaluating the MIC breakpoints specific to this formulation creates a significant gap in evidence-based guidance for its clinical use.

Given these challenges, this study was designed to investigate the MIC distribution of CPZ/SUL (1:1) in *P. aeruginosa* BSIs and to evaluate the association between MIC values and clinical outcomes. By examining the relationship between MIC levels and treatment response, this study aims to provide clinically relevant insights that may help inform antimicrobial decision-making.

## Materials and methods

### Study design, patient enrolment, and microbiological assessment

This retrospective multicentre study was conducted across eight Taiwanese medical centres and included patients diagnosed with *P. aeruginosa* BSIs between July 2017 and May 2024. The participating centres were Linkou Chang Gung Memorial Hospital (LCGMH), Kaohsiung Medical University Hospital (KMUH), Tri-Service General Hospital (TSGH), Taipei Veterans General Hospital (TVGH), China Medical University Hospital (CMUH), Taichung Veterans General Hospital (TCVGH), Chi Mei Medical Center (CMMC), and Kaohsiung Chang Gung Memorial Hospital (KCGMH). A total of 129 patients met the inclusion criteria: age ≥18 years, confirmed *P. aeruginosa* growth from blood cultures, and receipt of cefoperazone–sulbactam (CPZ/SUL) therapy initiated within 48 hours of bacteraemia onset. For the primary analysis, only patients who received CPZ/SUL therapy for at least three consecutive days were included to ensure adequate therapeutic exposure, and who had evaluable clinical outcomes were analysed. CPZ/SUL was administered according to institutional practice at the participating centres. The standard regimen consisted of CPZ/SUL 2 g/2 g intravenously every 12 h, administered as an intravenous infusion over approximately 30 min. Dose adjustments were performed in patients with renal impairment based on the treating physician’s clinical judgment and institutional dosing guidelines. Extended or prolonged infusion strategies were not used during the study period. For clarity and conciseness, CPZ/SUL hereafter refers to the 1:1 formulation unless otherwise specified.

In Taiwan, CPZ/SUL is used as a carbapenem-sparing agent for suspected or confirmed *P. aeruginosa* infections, particularly when the isolate is not known to be carbapenem-resistant or when antimicrobial stewardship programs aim to limit carbapenem use. The choice of CPZ/SUL was determined by the treating physicians in accordance with local clinical practice and established clinical practice guidelines, which generally take into account factors such as infection severity, suspected pathogens, antimicrobial stewardship principles, and patient-specific considerations.^12–14^ Difficult-to-treat resistance (DTR) is defined as *P. aeruginosa* exhibiting non-susceptibility to all of the following: piperacillin-tazobactam, ceftazidime, cefepime, imipenem-cilastatin and levofloxacin.^15^

**Figure 1.**
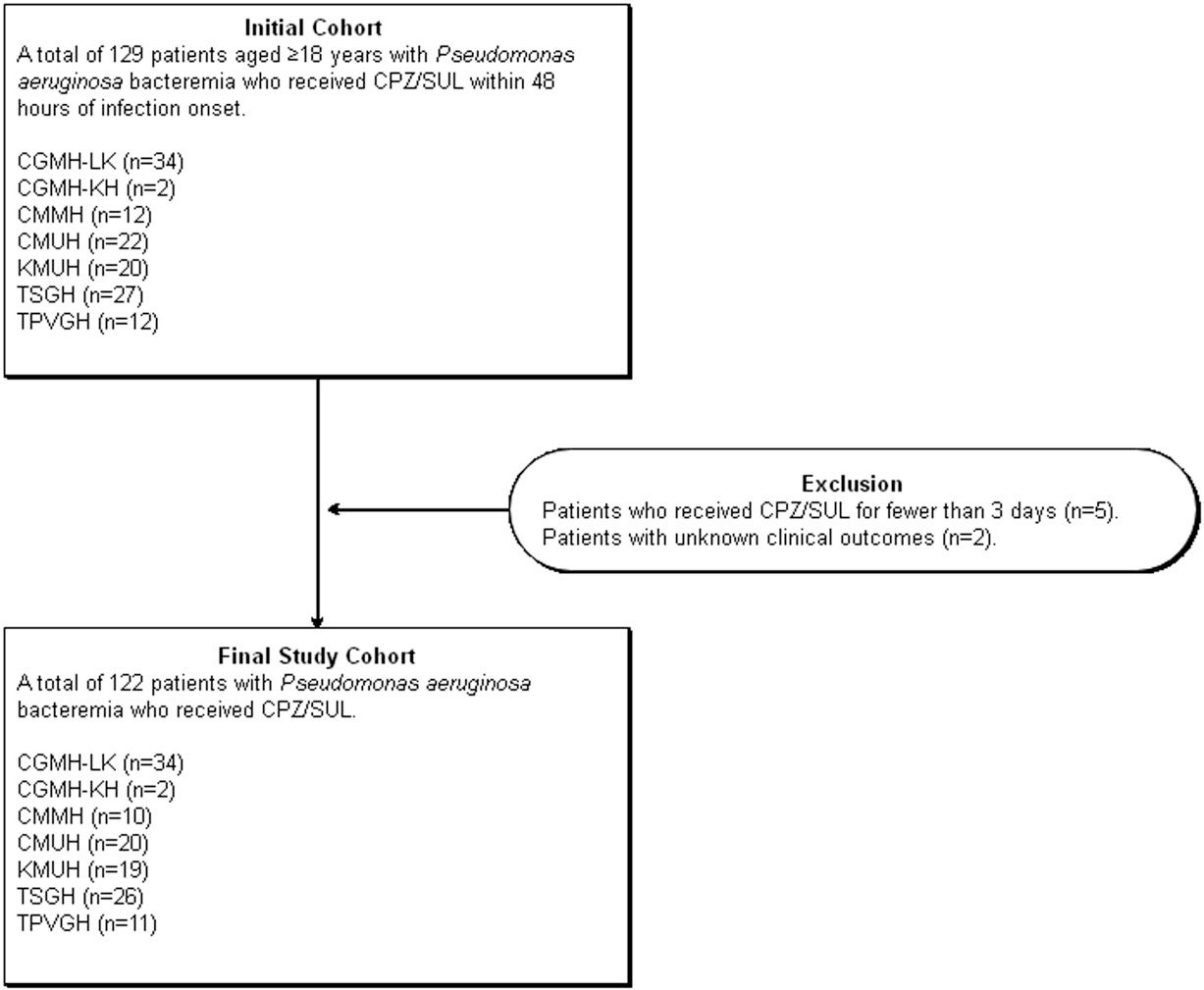
Flowchart illustrating the enrolment process of patients with *Pseudomonas aeruginosa* bloodstream infections treated with CPZ/SUL.

Exclusion criteria included unknown clinical outcomes, recurrent bacteraemia, polymicrobial BSIs, and concomitant use of antibiotics other than CPZ/SUL for more than 48 hours. After applying these criteria, 122 patients were included in the primary analysis. The detailed characteristics of excluded patients and reasons for exclusion are provided in Table S1 (available as Supplementary data at *JAC-AMR* Online). To address potential immortal-time bias, patients who received <3 days of CPZ/SUL therapy were also described separately, and a sensitivity analysis including these patients was performed when outcome data were available. The patient enrolment process of this study is detailed in Figure 1. The study was approved by the Institutional Review Board (IRB) of all of the participating hospitals.

All isolates were identified by the clinical microbiology laboratories of each participating hospital using matrix-assisted laser desorption/ionization time-of-flight mass spectrometry (MALDI-TOF MS) systems—either the Bruker Biotyper (Bruker Daltonics, Germany) or the VITEK^®^ MS (bioMérieux, France)—for species-level identification. For microbiological assessment, blood cultures were obtained from all included patients, and the isolated *P. aeruginosa* strains (*n* = 122) were subjected to antimicrobial susceptibility testing. To ensure methodological consistency, all susceptibility testing for this study was performed in a single central laboratory at Kaohsiung Medical University Hospital (KMUH). MICs for cefoperazone alone and for cefoperazone–sulbactam (CPZ/SUL, 1:1) were determined using the agar dilution method. Standard quality control (QC) strains were included in each testing run according to CLSI guidelines, including *P. aeruginosa* ATCC 27853, and QC results were verified to fall within the acceptable CLSI reference ranges before interpretation of clinical isolate results. These *P. aeruginosa* isolates were also tested against a panel of antibiotics, including amikacin, ceftazidime, cefepime, imipenem, levofloxacin, and piperacillin/tazobactam, in the central laboratory of KMUH using the VITEK 2 automated system (bioMérieux).

Clinical outcomes were determined through retrospective review of medical records by study investigators at each participating centre. Because MIC testing was performed in a central laboratory after isolate collection for study purposes, clinicians involved in routine patient care and outcome assessment were not aware of the study-specific MIC determinations. Clinical outcomes were assessed based on each patient's response at the end of therapy and categorized into two groups. Group 1 included patients with favourable outcomes classified as ‘cure’ (i.e. complete resolution of clinical symptoms and eradication of the pathogen) or ‘clinical improvement’ (i.e. significant symptom reduction without complete resolution). Group 2 included patients with poor outcomes classified as ‘lack of efficacy’ (i.e. failure to achieve clinical improvement or microbiological eradication) or ‘death’ (i.e. in-hospital mortality). Mortality was defined as all-cause mortality during the study period.

Key clinical variables, including age, gender, comorbidities, Charlson comorbidity index,^16^ prior intensive care unit (ICU) admission, presence of shock, severity scores, immunosuppressive therapy, infection sources and carbapenem resistance, were systematically collected for all patients. Immunosuppressive therapy was defined as the use of systemic corticosteroids (equivalent to ≥20 mg/day of prednisone for ≥2 weeks), chemotherapy, or immunosuppressive agents such as calcineurin inhibitors, antimetabolites, or biologics. Severity of illness was assessed using validated scoring systems, including the Acute Physiology and Chronic Health Evaluation II (APACHE II),^17^ Sequential Organ Failure Assessment (SOFA),^18^ and Pitt bacteraemia scores,^19^ ensuring a thorough stratification of disease severity. Infection sources were categorized into lung, urinary tract, intra-abdominal, catheter-related, and soft tissue infections, as well as primary bacteraemia. Diagnosis of each infection entity was based on standard clinical and microbiological criteria: pneumonia required compatible respiratory symptoms and new pulmonary infiltrates on imaging; urinary tract infection was defined by pyuria and a positive urine culture; intra-abdominal infection was confirmed by imaging or intraoperative findings with clinical signs of infection; catheter-related infection was diagnosed by clinical signs at the catheter site and either positive catheter tip culture or differential time to positivity; soft tissue infection was identified by localized inflammation with or without purulent drainage; and primary bacteraemia was assigned when no other infection focus could be identified after thorough evaluation.

### Statistical analysis

Continuous variables were summarized as mean ± standard deviation, while categorical variables were expressed as counts and percentages. Associations between clinical outcomes and sociodemographic, clinical characteristics, and the MICs of CPZ/SUL were assessed using Student’s *t*-test for continuous variables and the chi-square test for categorical variables. Univariate analyses were performed to evaluate associations between baseline clinical characteristics, microbiological factors (including CPZ/SUL MIC categories), and clinical outcomes. Variables with a *P*-value <0.10 in univariate analyses, along with clinically relevant variables selected *a priori*, were considered for inclusion in multivariable models. To identify independent predictors of poor clinical outcomes, multivariable analyses were conducted using generalized estimating equations (GEE) with a logit link function and an exchangeable working correlation structure to account for clustering of patients within participating hospitals. A sequential hierarchical modelling strategy was applied. Model 1 evaluated the crude association between CPZ/SUL MIC (≥32 mg/L versus ≤16 mg/L) and clinical outcomes. Model 2 adjusted for demographic characteristics and comorbidity burden, including age, gender, immunosuppressive therapy, and Charlson comorbidity index. Model 3 (fully adjusted model) further incorporated acute clinical variables and disease severity indices, including shock, prior ICU admission, Pitt bacteraemia score, and SOFA score. Additional clinically relevant variables, including carbapenem resistance and infection source, were further included in the fully adjusted model. Adjusted odds ratios (aORs) with 95% confidence intervals (CIs) were reported. To enhance clinical interpretability, adjusted probabilities and risk differences were estimated from the fully adjusted model using marginal standardization based on the fully adjusted GEE model. To assess the robustness of the primary findings and address potential immortal-time bias associated with the ≥3-day treatment requirement, a sensitivity analysis including patients who received <3 days of CPZ/SUL therapy was performed. Missing data were not observed for primary exposures or key covariates; therefore, no imputation was performed. All statistical analyses were performed using SAS software version 9.4 (SAS Institute, Cary, NC, USA). A two-tailed *P*-value of <0.05 was considered statistically significant.

## Results

### Associations between clinical factors and outcomes

Among the 122 patients with *P. aeruginosa* BSI treated with CPZ/SUL at a 1:1 ratio, 93 (76.2%) had a favourable clinical response. In the univariate analysis (Table 1), baseline demographic characteristics—including age, gender, and comorbid conditions (liver or renal impairment, heart failure, diabetes mellitus, neutropenia, malignancy, immunosuppressive therapy, and Charlson comorbidity index)—did not differ significantly between patients with favourable and poor outcomes. However, markers of acute illness severity were notably higher in the poor outcome group: a greater proportion had Pitt bacteraemia scores >4 (20.7% versus 7.5%, *P* = 0.045), and mean APACHE II (17.8 ± 8.7 versus 14.1 ± 7.2; *P* = 0.020) and SOFA scores (5.9 ± 3.8 versus 3.4 ± 3.2; *P* < 0.001) were elevated. With regard to microbiological characteristics, isolates with CPZ/SUL MIC ≥32 mg/L were more frequently observed in the poor outcome group compared with the favourable outcome group (34.5% versus 12.9%; *P* = 0.029). In addition, carbapenem-resistant *P. aeruginosa* (CRPA) was more commonly identified among patients with poor outcomes, although this difference did not reach statistical significance. Conversely, infections caused by isolates with lower MIC values (≤16 mg/L) were more commonly associated with favourable outcomes.

**Table 1.** Summary of the univariate analysis of baseline demographics, clinical parameters, disease severity, and microbiological factors in the favourable versus poor outcome groups

	Poor outcome (*n* = 29)		Favourable outcome (*n* = 93)		
	*n*	%	*n*	%	*P*-value
Gender (male)	22	75.9	63	67.7	0.406
Age (years ± SD)	66.2 ± 15.5		65.5 ± 15.5		0.828
Co-morbidities					
Liver function impairment	5	17.2	8	8.6	0.188
Renal function impairment	6	20.7	20	21.5	0.925
Heart failure	5	17.2	11	11.8	0.451
Diabetes mellitus	5	17.2	25	26.9	0.293
Neutropenia	1	3.5	3	3.2	0.953
Immunosuppressive therapy	1	3.5	6	6.5	0.544
Malignancy	16	55.2	44	47.3	0.460
Charlson score					
None (score = 0)	1	3.5	3	3.2	
Mild (score = 1–2)	1	3.5	9	9.7	
Moderate (score = 3–4)	10	34.4	22	23.7	
Severe (score ≥5)	17	58.6	59	63.4	
Mean ± SD	6.2 ± 3.3		5.7 ± 2.7		0.463
Shock	3	10.3	10	10.8	0.950
Previous ICU admission	5	17.2	12	12.9	0.556
Pitt bacteraemia score >4	6	20.7	7	7.5	0.045*
APACHE II score					
0–10	6	20.7	30	32.3	
11–20	14	48.3	47	50.5	
21–30	7	24.1	14	15.0	
>30	2	6.9	2	2.2	
Mean ± SD	17.8 ± 8.7		14.1 ± 7.2		0.020*
SOFA score					
0–6	17	58.6	75	80.7	
7–9	6	20.7	14	15.0	
10–12	5	17.2	3	3.2	
≥13	1	3.5	1	1.1	
Mean ± SD	5.9 ± 3.8		3.4 ± 3.2		<0.001*
MIC (CPZ/SUL 1:1)					0.029*
≤16 mg/L	19	65.5	81	87.1	
≥32 mg/L	10	34.5	12	12.9	
Carbapenem resistance (CRPA^a^)	9	31.0	13	14.0	0.037*

^a^Carbapenem-resistant *P. aeruginosa*.

* *P* < 0.05.

The results of the multivariable analysis using GEE, accounting for clustering by study centre, are presented in Table 2. Across all three hierarchical models, an MIC ≥32 mg/L remained significantly associated with an increased risk of poor clinical outcomes. In the unadjusted model (Model 1), MIC ≥32 mg/L was associated with a higher likelihood of poor outcomes (OR 3.12, 95% CI 1.78–5.45, *P* < 0.001). This association remained significant after adjustment for demographic characteristics and comorbidity burden in Model 2 (aOR 3.22, 95% CI 1.50–6.92, *P* = 0.003), and persisted in the fully adjusted model (Model 3), which further incorporated acute clinical variables, disease severity indices, carbapenem resistance, and pneumonia (aOR 2.74, 95% CI 1.44–5.25, *P* = 0.002). In the fully adjusted model, male sex, higher Charlson comorbidity index (>2), and higher SOFA scores were independently associated with an increased risk of poor clinical outcomes, whereas carbapenem resistance and pneumonia were not significantly associated with outcomes. Given the lack of statistical significance in the fully adjusted model and the relatively limited number of cases within these subgroups, no further stratified analyses (e.g. MIC-based subgroup comparisons within CRPA or pneumonia) were performed.

**Table 2.** Univariate and multivariable analyses of factors associated with poor clinical outcomes in *P. aeruginosa* bloodstream infections treated with CPZ/SUL using generalized estimating equations (GEE) accounting for clustering by study centre

	Model 1			Model 2			Model 3		
	OR	95% CI	*P*-value	aOR	95% CI	*P*-value	aOR	95% CI	*P*-value
MIC ≥32 mg/L (1:1)	3.12	(1.78, 5.45)	<0.001*	3.22	(1.50, 6.92)	0.003*	2.74	(1.44, 5.25)	0.002*
Male				1.80	(1.19, 2.73)	0.005*	1.91	(1.31, 2.79)	<0.001*
Age (per year)				0.99	(0.96, 1.01)	0.366	0.98	(0.96, 1.01)	0.138
Immunosuppressant therapy				0.39	(0.09, 1.65)	0.199	0.45	(0.20, 1.03)	0.058
Charlson comorbidity index >2				3.48	(1.47, 8.26)	0.005*	3.61	(1.11, 11.73)	0.033*
Shock							0.52	(0.15, 1.73)	0.282
Previous ICU admission							0.51	(0.18, 1.45)	0.204
Pitt bacteraemia score >4							0.93	(0.46, 1.88)	0.841
SOFA score							1.23	(1.06, 1.42)	0.005*
CRPA^a^							0.96	(0.33, 2.79)	0.942
Pneumonia (versus non-pneumonia)							2.00	(1.00, 4.00)	0.051

Model 1, unadjusted model; Model 2, adjusted for demographic characteristics and comorbidity burden (age, gender, immunosuppressive therapy, and Charlson comorbidity index); Model 3, fully adjusted model additionally incorporating acute clinical variables and disease severity indices (shock, prior ICU admission, Pitt bacteraemia score, and SOFA score), as well as carbapenem resistance and infection source (pneumonia).

^a^Carbapenem-resistant *P. aeruginosa*.

^*^ *P* < 0.05.

To provide clinically interpretable effect estimates, adjusted risks and risk differences for poor clinical outcomes according to MIC categories were further estimated (Table 3). After adjustment for potential confounders, patients infected with isolates with MIC ≥32 mg/L had a higher adjusted risk of poor outcomes compared with those with MIC ≤16 mg/L (34.9% versus 19.9%), corresponding to an adjusted risk difference of 15.0%. A sensitivity analysis including all evaluable patients who initiated CPZ/SUL therapy (*n* = 127) yielded results consistent with those of the primary analysis, with similar distributions of clinical outcomes (Table S2). These findings support the robustness of the primary results with respect to the ≥3-day treatment requirement.

**Table 3.** Adjusted risk differences of poor outcomes by MIC group in *P. aeruginosa* bloodstream infections treated with CPZ/SUL

MIC (mg/L)	*n*	Poor outcome (*n*)	Crude risk (%)	Adjusted risk (%)	Adjusted risk difference
≤16	100	19	19.0%	19.9%	Ref
≥32	22	10	45.4%	34.9%	15.0%

Adjusted risks and risk differences were estimated using marginal standardization based on the fully adjusted multivariable model (Model 3), which incorporated all prespecified covariates. ‘Ref’ indicates the reference group.

### Infection source and clinical outcomes

**Figure 2.**
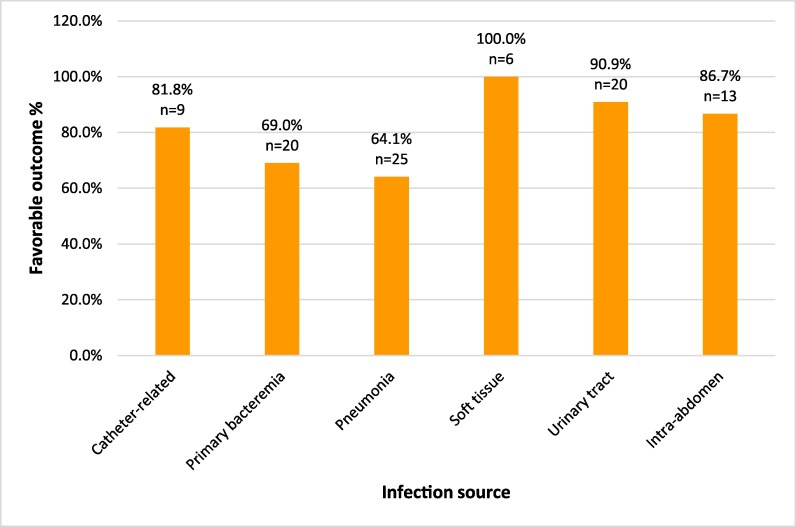
Comparison of infection sources stratified by clinical outcomes, highlighting the prognostic variability across different infection sites.

The comparison of infection sources by clinical outcomes demonstrates significant variability in prognostic implications across different infection sites (Figure 2). Favourable outcomes were most prevalent in soft tissue infections (100%), urinary tract infections (90.9%), and intra-abdominal infections (86.7%), highlighting their relatively better clinical responses. In contrast, pneumonia (64.1%) and primary bacteraemia (69.0%) were associated with poorer outcomes, reflecting the increased risks and challenges in managing these infection sources. These findings indicate that clinical outcomes varied according to the source of infection.

The associations between infection sources and clinical outcomes are summarized in Table 4. In the comparative analysis, pneumonia was more frequently observed among patients with poor outcomes than among those with favourable outcomes, and this difference reached statistical significance (48.3% versus 30.1%, *P* = 0.041). In contrast, other infection sources—including catheter-related infections (6.9% versus 9.7%, *P* = 0.999), primary bacteraemia (31.0% versus 21.5%, *P* = 0.397), and urinary tract infections (6.9% versus 21.5%, *P* = 0.099)—were not significantly associated with clinical outcomes. Notably, urinary tract infections were numerically less frequent in the poor outcome group, suggesting a trend towards more favourable outcomes, although this did not reach statistical significance. When considered in the context of multivariable analysis, however, the association observed for pneumonia was not sustained after adjustment, suggesting that the univariate finding may have been influenced by confounding factors.

**Table 4.** Clinical outcomes of patients with *P. aeruginosa* bacteraemia stratified by source of infection

Infections sources	Poor outcome (*n* = 29), *n* (%)	Favourable outcome (*n* = 93), *n* (%)	Difference	*P*-value
Catheter-related	2 (6.9)	9 (9.7)	−2.8%	0.999
Primary bacteraemia	9 (31.0)	20 (21.5)	+9.5%	0.397
Pneumonia	14 (48.3)	25 (26.9)	+21.4%	0.041*
Soft tissue	0 (0)	6 (6.5)	−6.5%	0.174
Urinary tract	2 (6.9)	20 (21.5)	−14.6%	0.099
Intra-abdomen	2 (6.9)	13 (14.0)	−7.1%	0.357

*P < 0.05.

### MIC distribution and clinical response

**Figure 3.**
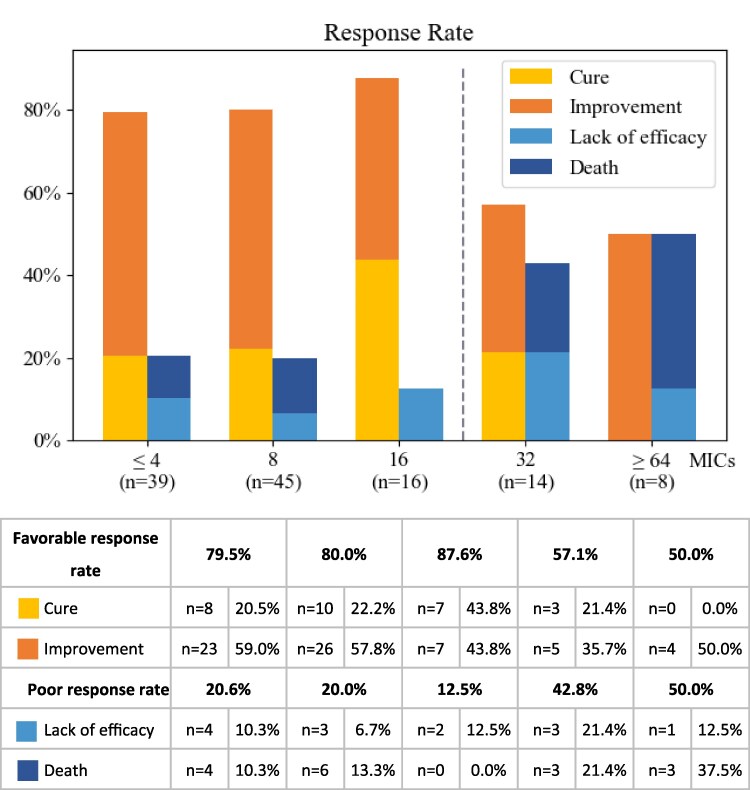
MIC values of CPZ/SUL (1:1) and clinical outcomes in patients received treatment with this antimicrobial agent. The favourable response rate—defined as the proportion of patients achieving cure or clinical improvement—was significantly higher among those with MICs ≤16 mg/L compared to those with MICs ≥32 mg/L (81% versus 54.5% favourable outcomes; OR = 3.55; 95% CI 1.34–9.43; *P* = 0.013).

A total of 122 *P. aeruginosa* isolates were tested for susceptibility to CPZ/SUL at a 1:1 ratio using the agar dilution method. The distribution of clinical outcomes according to MIC values is shown in Figure 3. Overall, higher rates of favourable clinical outcomes were observed among patients infected with isolates exhibiting lower MIC values. Favourable outcome rates were 79.5% for MIC ≤4 mg/L, 80.0% for MIC = 8 mg/L, and 87.6% for MIC = 16 mg/L, compared with 57.1% for MIC = 32 mg/L and 50.0% for MIC ≥64 mg/L. When MIC values were dichotomized, patients infected with isolates with MIC ≤16 mg/L had a higher proportion of favourable outcomes compared with those with MIC ≥32 mg/L (81.0% versus 54.5%). This difference was statistically significant (OR, 3.55; 95% CI, 1.34–9.43; *P* = 0.013), indicating an association between lower MIC values and improved clinical outcomes.

According to the 2022 Infectious Diseases Society of America (IDSA) criteria for difficult-to-treat resistance (DTR), only one isolate (0.8%) met the definition of DTR in this cohort.^15^ Given the extremely limited number of DTR cases, no further comparative analysis was performed.

### Antimicrobial susceptibility

Among the 122 *P. aeruginosa* isolates, the proportion of isolates with CPZ/SUL MIC ≤16 mg/L was 82%, based on a study-defined threshold using agar dilution. Table 5 summarizes the susceptibility profiles of additional antibiotics as assessed by the VITEK 2 automated system. Susceptibility rates varied across agents, with piperacillin–tazobactam demonstrating a lower susceptibility rate (74.6%) compared with most other tested antibiotics. CRPA accounted for 18% of the isolates. Among patients with CRPA infections who received CPZ/SUL (1:1) treatment, favourable clinical outcomes were observed in 59.1% of cases. Given the limited sample size and the lack of statistical significance in multivariable analysis (Table 2), these findings are presented descriptively.

**Table 5.** Antimicrobial susceptibility profiles of 122 *Pseudomonas aeruginosa* isolates (VITEK 2 automated system)

Antibiotic	Susceptible (%)	Intermediate (%)	Resistant (%)
Amikacin	97.5	0.8	1.7
Ceftazidime	85.2	4.1	10.7
Cefepime	86.9	10.7	2.5
Imipenem	82.0	0	18.0
Levofloxacin	82.0	6.6	11.5
Piperacillin/Tazobactam	74.6	8.2	17.2

### Comparison of cefoperazone alone and CPZ/SUL (1:1) MICs

Cefoperazone MICs determined by agar dilution for the 122 *P. aeruginosa* isolates are summarized in Table 6. Using the interpretive criteria referenced to CLSI (2023),^5^ 89/122 isolates (73%) were categorized as susceptible (MIC ≤16 mg/L), 14/122 (11%) intermediate (MIC =32 mg/L), and 19/122 (16%) resistant (MIC ≥64 mg/L). In the same set of isolates, CPZ/SUL (1:1) demonstrated a higher proportion of isolates with MIC ≤16 mg/L (100/122, 82%), based on the study-defined threshold. Overall, a greater proportion of isolates met the ≤16 mg/L threshold for CPZ/SUL compared with cefoperazone alone (82% versus 73%).

**Table 6.** MIC distribution of *Pseudomonas aeruginosa* isolates (*n* = 122) for cefoperazone alone and for cefoperazone-sulbactam (1:1) as determined by agar dilution

Antimicrobial agent	≤16 (S)	=32 (I)	≥64 (R)	Total
Cefoperazone	89 (73%)	14 (11%)	19 (16%)	122 (100%)
Cefoperazone/ Sulbactam (1:1)	100 (82%)	14 (11%)	8 (7%)	122 (100%)

Interpretive categories were assigned according to CLSI (2023) criteria for cefoperazone: S, susceptible (≤16 mg/L); I, intermediate (=32 mg/L); R, resistant (≥64 mg/L).

## Discussion

### Association between MIC values and clinical outcomes

In this multicentre cohort, we observed a consistent and clinically meaningful association between CPZ/SUL MIC values and treatment outcomes in patients with *P. aeruginosa* BSIs. Across multiple analytical approaches—including univariate comparisons, multivariable modelling, and adjusted risk estimation—higher MIC values were associated with an increased likelihood of poor clinical outcomes. In the univariate analysis, isolates with MIC ≥32 mg/L were significantly more frequent among patients with poor outcomes, alongside established markers of disease severity such as higher SOFA and APACHE II scores. This suggests that both host factors and microbiological characteristics may contribute to outcome variability. However, importantly, the association between elevated MIC values and poor outcomes persisted after adjustment for these confounders. In multivariable analysis using GEEs to account for inter-centre clustering, an MIC ≥32 mg/L remained independently associated with poor outcomes across all hierarchical models. This association was robust to adjustment for demographic factors, comorbidity burden, and severity of illness, indicating that MIC is not merely a surrogate for baseline patient severity but represents an independent predictor of treatment response. To translate this association into clinically interpretable effect estimates, patients with MIC ≥32 mg/L had a higher adjusted risk of poor outcomes than those with MIC ≤16 mg/L (34.9% versus 19.9%), corresponding to an absolute risk difference of 15.0%. While these findings underscore the potential clinical importance of MIC stratification, they should not be interpreted as establishing a formal clinical breakpoint. Instead, they provide outcome-based evidence that may inform antimicrobial stewardship and individualized treatment decisions.

### Comparison with previous studies and interpretation

Previous studies have evaluated the activity of CPZ/SUL against a variety of Gram-negative pathogens and infection types.^20–24^ However, clinical data specifically examining the relationship between CPZ/SUL MIC values and treatment outcomes in *P. aeruginosa* BSIs remain limited. The association observed in this study between lower MIC values and improved clinical outcomes is consistent with prior *in vitro* susceptibility studies reporting that a substantial proportion of *P. aeruginosa* isolates remain susceptible to CPZ/SUL at MIC values ≤16 mg/L.^1^ Taken together, these findings provide clinical context for interpreting CPZ/SUL MIC values in patients with *P. aeruginosa* BSIs. While the observed MIC–outcome relationship is clinically informative, its mechanistic basis cannot be fully elucidated within the scope of this study.

### Clinical relevance in CRPA infections

The potential role of CPZ/SUL is relevant in the context of increasing MDR *P. aeruginosa*, including CRPA.^4,9,25,26^ In this study, CPZ/SUL therapy was associated with favourable outcomes in 59.1% of CRPA cases. Although this observation should be interpreted cautiously due to the limited number of cases, it suggests that CPZ/SUL may still provide clinical benefit in selected infections caused by carbapenem-resistant isolates. This observation is consistent with prior *in vitro* and treatment-based studies demonstrating that CPZ/SUL retains activity against MDR Gram-negative organisms.^27–30^ Because resistance mechanisms in CRPA are highly heterogeneous, including β-lactamase production, efflux pump overexpression, and porin alterations, the clinical activity of CPZ/SUL likely varies across resistance profiles. Future studies incorporating molecular characterization of resistance determinants are warranted to better define the role of CPZ/SUL in specific CRPA subgroups.

### Pharmacokinetic and pharmacodynamic considerations

Interpretation of MIC–outcome relationships for β-lactam antibiotics should be grounded in PK/PD principles, particularly the duration for which free drug concentrations exceed the MIC (%fT>MIC). Based on previously reported pharmacokinetic data, serum concentrations of cefoperazone following intravenous administration (2 g) exceed 16 mg/L shortly after infusion and remain above this level for several hours in patients with normal renal function, with more prolonged exposure in those with impaired renal function.^31^ These findings support the pharmacodynamic plausibility that standard CPZ/SUL dosing (2 g/2 g every 12 hours with conventional short infusion) can achieve adequate %fT>MIC for isolates with MIC values ≤16 mg/L, consistent with the clinical associations observed in this study. Nevertheless, this interpretation is based on total drug concentrations and descriptive pharmacokinetic data rather than formal PK/PD modelling, and should therefore be interpreted with caution. In clinical practice, target attainment may vary according to patient-specific factors, including renal function and augmented renal clearance. Alternative dosing strategies, such as prolonged or extended infusions, may further optimize %fT>MIC and could be considered, particularly for isolates with higher MIC values.

### Interpretation of mortality outcomes

In this cohort, 16 patients died during the study period. Mortality was assessed as all-cause mortality, as attribution specifically to *P. aeruginosa* BSI was not feasible due to the complex clinical conditions and comorbidities of the study population. In observational studies of BSIs, distinguishing infection-attributable mortality from underlying disease-related mortality is inherently challenging. Therefore, all-cause mortality represents a pragmatic and objective outcome measure, although it may not fully reflect infection-specific effects.

### Implications for clinical practice and antimicrobial stewardship

From a clinical perspective, the observed association between CPZ/SUL MIC values and treatment outcomes may have implications for antimicrobial stewardship and therapeutic decision-making. Lower MIC values may indicate a higher likelihood of favourable response when CPZ/SUL is used for the treatment of *P. aeruginosa* infections. In this cohort, the majority of isolates remained susceptible to imipenem (82%), reflecting a relatively low prevalence of DTR *P. aeruginosa*. CPZ/SUL is used in clinical practice as a carbapenem-sparing alternative, particularly when adequate source control is achieved and isolates exhibit low-to-intermediate MIC values. In many clinical settings, such prescribing practices are guided by antimicrobial stewardship strategies aimed at preserving carbapenem efficacy and reducing selective pressure for resistant organisms.

### Strengths and contributions

This study is among the first to focus specifically on the 1:1 CPZ/SUL formulation in the treatment of *P. aeruginosa* BSIs, offering MIC-stratified analysis of clinical outcomes. Most previous investigations on CPZ/SUL and *P. aeruginosa* have been limited to *in vitro* susceptibility studies, with few addressing the correlation between MIC values and clinical effectiveness.^1,9,11^ By characterizing the association between MIC levels and treatment response, our findings help address this gap and offer clinically relevant insights that may inform antimicrobial decision-making and stewardship strategies.

### Limitations and future directions

Despite its strengths, this study has several limitations. First, the retrospective design limits the ability to control for treatment selection bias, and the inclusion of eight tertiary-care centres introduces potential inter-institutional heterogeneity in both patient characteristics and microbiological testing. Nonetheless, standardized inclusion criteria and centralized susceptibility verification reduced such variability. In addition, the retrospective nature of the study precludes causal inference between antimicrobial therapy and clinical outcomes.

Another limitation relates to potential indication bias. The decision to prescribe CPZ/SUL was made by treating physicians and may have been influenced by clinical severity, infection source, or antimicrobial stewardship considerations. Because a comparator cohort of patients treated with carbapenems or other antipseudomonal agents was not included, it is not possible to directly evaluate whether patients selected for CPZ/SUL differed systematically from those receiving alternative therapies.

A further limitation relates to the inclusion criterion requiring ≥3 days of CPZ/SUL therapy. This approach was adopted to ensure adequate antimicrobial exposure for outcome evaluation; however, it may introduce immortal-time bias by excluding patients who died early or discontinued therapy shortly after treatment initiation. To address this concern, excluded cases were reported, and a sensitivity analysis including patients who received <3 days of therapy was performed when outcome data were available. The findings of this analysis were generally consistent with the primary results, suggesting that the overall conclusions are unlikely to be substantially affected by this potential bias.

Finally, molecular resistance profiling was not systematically performed in this study, which limited the ability to fully elucidate the mechanistic basis underlying the observed MIC shifts between cefoperazone alone and CPZ/SUL. Future investigations incorporating prospective study designs, pharmacokinetic/pharmacodynamic modeling, and integrated genomic or phenotypic resistance characterization with MIC–outcome correlations are warranted to refine CPZ/SUL breakpoints and to assess the consistency of these associations across different resistance mechanisms.

### Conclusion

In this multicentre cohort, lower CPZ/SUL MIC values (≤16 mg/L) were associated with improved outcomes in patients with *P. aeruginosa* BSIs. Outcomes appeared to vary by infection source, with pneumonia generally associated with less favourable responses, highlighting the importance of clinical context when interpreting treatment response. These findings provide clinically relevant evidence to support MIC-guided therapeutic decision-making and antimicrobial stewardship.

## Supplementary Material

dlag073_Supplementary_Data

## Supplementary data

Tables S1 and S2 are available as Supplementary data at *JAC-AMR* Online.

## References

[1] Sader HS, Carvalhaes CG, Streit JM et al Antimicrobial activity of cefoperazone-sulbactam tested against gram-negative organisms from Europe, Asia-Pacific, and Latin America. Int J Infect Dis 2020; 91: 32–7. 10.1016/j.ijid.2019.11.006 31715325

[2] Babich T, Naucler P, Valik JK et al Risk factors for mortality among patients with Pseudomonas aeruginosa bacteraemia: a retrospective multicentre study. Int J Antimicrob Agents 2020; 55: 105847. 10.1016/j.ijantimicag.2019.11.004 31770625

[3] Kim YJ, Jun YH, Kim YR et al Risk factors for mortality in patients with Pseudomonas aeruginosa bacteremia; retrospective study of impact of combination antimicrobial therapy. BMC Infect Dis 2014; 14: 161. 10.1186/1471-2334-14-161 24655422 PMC3994322

[4] Li Y, Liu X, Yao H et al The evolution of carbapenem-resistant Pseudomonas aeruginosa in the COVID-19 era: a global perspective and regional insights. Int J Antimicrob Agents 2025; 65: 107466. 10.1016/j.ijantimicag.2025.107466 39971140

[5] Clinical and Laboratory Standards Institute (CLSI) . *Performance standards for antimicrobial susceptibility testing—Thirty-Five Edition: M100*. 2023.

[6] Bodey GP, Miller P, Ho DH. In vitro assessment of sulbactam plus cefoperazone in the treatment of bacteria isolated from cancer patients. Diagn Microbiol Infect Dis 1989; 12(Suppl 4): S209–14. 10.1016/0732-8893(89)90138-7 2591178

[7] Niu T, Luo Q, Li Y et al Comparison of Tigecycline or Cefoperazone/Sulbactam therapy for bloodstream infection due to carbapenem-resistant Acinetobacter baumannii. Antimicrob Resist Infect Control 2019; 8: 52. 10.1186/s13756-019-0502-x 30886705 PMC6404342

[8] Jones RN, Barry AL, Packer RR et al In vitro antimicrobial spectrum, occurrence of synergy, and recommendations for dilution susceptibility testing concentrations of the cefoperazone-sulbactam combination. J Clin Microbiol 1987; 25: 1725–9. 10.1128/jcm.25.9.1725-1729.1987 3498740 PMC269316

[9] Lai CC, Chen CC, Lu YC et al In vitro activity of cefoperazone and cefoperazone-sulbactam against carbapenem-resistant Acinetobacter baumannii and Pseudomonas aeruginosa. Infect Drug Resist 2019; 12: 25–9. 10.2147/IDR.S181201 30588045 PMC6304247

[10] Chang PC, Chen CC, Lu YC et al The impact of inoculum size on the activity of cefoperazone-sulbactam against multidrug resistant organisms. J Microbiol Immunol Infect 2018; 51: 207–13. 10.1016/j.jmii.2017.08.026 29037802

[11] Sheu MJ, Chen CC, Lu YC et al In vitro antimicrobial activity of Various cefoperazone/sulbactam products. Antibiotics (Basel) 2020; 9: 77. 10.3390/antibiotics9020077 32059590 PMC7168170

[12] Chou CC, Shen CF, Chen SJ et al Recommendations and guidelines for the treatment of pneumonia in Taiwan. J Microbiol Immunol Infect 2019; 52: 172–99. 10.1016/j.jmii.2018.11.004 30612923

[13] Gomi H, Solomkin JS, Schlossberg D et al Tokyo guidelines 2018: antimicrobial therapy for acute cholangitis and cholecystitis. J Hepatobiliary Pancreat Sci 2018; 25: 3–16. 10.1002/jhbp.518 29090866

[14] Mazuski JE, Tessier JM, May AK et al The Surgical Infection Society revised guidelines on the management of intra-abdominal infection. Surg Infect (Larchmt) 2017; 18: 1–76. 10.1089/sur.2016.261 28085573

[15] Tamma PD, Aitken SL, Bonomo RA et al Infectious Diseases Society of America 2022 guidance on the treatment of extended-Spectrum beta-lactamase producing enterobacterales (ESBL-E), carbapenem-resistant enterobacterales (CRE), and Pseudomonas aeruginosa with difficult-to-treat resistance (DTR-P. aeruginosa). Clin Infect Dis 2022; 75: 187–212. 10.1093/cid/ciac268 35439291 PMC9890506

[16] Charlson ME, Pompei P, Ales KL et al A new method of classifying prognostic comorbidity in longitudinal studies: development and validation. J Chronic Dis 1987; 40: 373–83. 10.1016/0021-9681(87)90171-8 3558716

[17] Knaus WA, Draper EA, Wagner DP et al APACHE II: a severity of disease classification system. Crit Care Med 1985; 13: 818–29. 10.1097/00003246-198510000-00009 3928249

[18] Vincent JL, Moreno R, Takala J et al The SOFA (sepsis-related organ failure assessment) score to describe organ dysfunction/failure. On behalf of the working group on sepsis-related problems of the European Society of Intensive Care Medicine. Intensive Care Med 1996; 22: 707–10. 10.1007/BF01709751 8844239

[19] Hilf M, Yu VL, Sharp J et al Antibiotic therapy for Pseudomonas aeruginosa bacteremia: outcome correlations in a prospective study of 200 patients. Am J Med 1989; 87: 540–6. 10.1016/S0002-9343(89)80611-4 2816969

[20] Chen RZ, Lu PL, Yang TY et al Efficacy of cefoperazone/sulbactam for ESBL-producing Escherichia coli and Klebsiella pneumoniae bacteraemia and the factors associated with poor outcomes. J Antimicrob Chemother 2024; 79: 648–55. 10.1093/jac/dkae022 38319833

[21] Lai CC, Chen WC, Kuo LK et al The clinical efficacy of cefoperazone-sulbactam versus piperacillin-tazobactam in the treatment of severe community-acquired pneumonia. Medicine (Baltimore) 2023; 102: e34284. 10.1097/MD.0000000000034284 37443505 PMC10344575

[22] Wang Q, Huang M, Zhou S. Observation of clinical efficacy of the cefoperazone/sulbactam anti-infective regimen in the treatment of multidrug-resistant Acinetobacter baumannii lung infection. J Clin Pharm Ther 2022; 47: 1020–7. 10.1111/jcpt.13638 35285526

[23] Chen CH, Tu CY, Chen WC et al Clinical efficacy of cefoperazone-sulbactam versus piperacillin-tazobactam in the treatment of hospital-acquired pneumonia and ventilator-associated pneumonia. Infect Drug Resist 2021; 14: 2251–8. 10.2147/IDR.S313828 34168466 PMC8216753

[24] Liu JW, Chen YH, Lee WS et al Randomized noninferiority trial of cefoperazone-sulbactam versus cefepime in the treatment of hospital-acquired and healthcare-associated pneumonia. Antimicrob Agents Chemother 2019; 63: e00023-19. 10.1128/AAC.00023-19 31138577 PMC6658783

[25] Yang F, Zhao Q, Wang L et al Diminished susceptibility to Cefoperazone/Sulbactam and Piperacillin/Tazobactam in Enterobacteriaceae due to narrow-Spectrum beta-lactamases as well as omp mutation. Pol J Microbiol 2022; 71: 251–6. 10.33073/pjm-2022-023 35716168 PMC9252146

[26] Pfaller MA, Flamm RK, Duncan LR et al Antimicrobial activity of tigecycline and cefoperazone/sulbactam tested against 18,386 gram-negative organisms from Europe and the Asia-Pacific region (2013-2014). Diagn Microbiol Infect Dis 2017; 88: 177–83. 10.1016/j.diagmicrobio.2017.02.020 28341098

[27] Li Y, Xie J, Chen L et al Treatment efficacy of tigecycline in comparison to cefoperazone/sulbactam alone or in combination therapy for carbapenenm-resistant Acinetobacter baumannii infections. Pak J Pharm Sci 2020; 33: 161–8. 10.36721/PJPS.2020.33.1.REG.161-168.1 32122844

[28] Li T, Sheng M, Gu T et al In vitro assessment of cefoperazone-sulbactam based combination therapy for multidrug-resistant Acinetobacter baumannii isolates in China. J Thorac Dis 2018; 10: 1370–6. 10.21037/jtd.2018.02.01 29707286 PMC5906317

[29] Cai Y, Yang D, Wang J et al Amikacin and cefoperazone/sulbactam alone or in combination against carbapenem-resistant Pseudomonas aeruginosa. Diagn Microbiol Infect Dis 2018; 91: 186–90. 10.1016/j.diagmicrobio.2018.01.023 29486972

[30] Liu B, Bai Y, Liu Y et al In vitro activity of tigecycline in combination with cefoperazone-sulbactam against multidrug-resistant Acinetobacter baumannii. J Chemother 2015; 27: 271–6. 10.1179/1973947814Y.0000000203 25068186

[31] Reitberg DP, Marble DA, Schultz RW et al Pharmacokinetics of cefoperazone (2.0g) and sulbactam (1.0g) coadministered to subjects with normal renal function, patients with decreased renal function, and patients with end-stage renal disease on hemodialysis. Antimicrob Agents Chemother 1988; 32: 503–9. 10.1128/AAC.32.4.503 3377461 PMC172210

