# Supplementary material for: Clinical implications of cefoperazone–sulbactam MIC values in Pseudomonas aeruginosa bloodstream infections: a multicentre study
Source: JAC Antimicrob Resist. 2026 May 11;8(3):dlag073. doi: 10.1093/jacamr/dlag073 (PMC13158015; doi:10.1093/jacamr/dlag073)
Supplement: dlag073_Supplementary_Data [file dlag073_supplementary_data.docx]

| Reasons for exclusion | Number | Comment |
| --- | --- | --- |
| CPZ/SUL therapy <3 days | 5 |  |
| Unknown clinical outcome | 2 | Transferred out; outcome unavailable |
| Polymicrobial bacteremia | 0 |  |
| Recurrent bacteremia | 0 |  |
| Concomitant antibiotics other than CPZ/SUL  for >48 h | 0 |  |
| Total excluded | 7 |  |

Supplementary Table 1. Detailed reasons for exclusion of patients from the final analytic cohort.

| Cohort | Number | Favorable outcome | Poor outcome | p-value † |
| --- | --- | --- | --- | --- |
| ≥3 days CPZ/SUL (primary analysis) | 122 | 93 (76.2%) | 29 (23.8%) |  |
| All evaluable CPZ/SUL patients* | 127 | 95 (74.8%) | 32 (25.2%) | 0.79 |

Supplementary Table 2. Clinical outcomes in the primary cohort and the expanded cohort including patients receiving <3 days of CPZ/SUL therapy.

*Excluding two patients with unknown clinical outcomes.

†P value calculated using the chi-square test comparing the distribution of favorable and poor outcomes between the two cohorts.
